# Distinct patterns of personalised dietary advice delivered by a metabotype framework similarly improve dietary quality and metabolic health parameters: secondary analysis of a randomised controlled trial

**DOI:** 10.3389/fnut.2023.1282741

**Published:** 2023-11-15

**Authors:** Elaine Hillesheim, Lorraine Brennan

**Affiliations:** ^1^UCD School of Agriculture and Food Science, Institute of Food and Health, University College Dublin, Dublin, Ireland; ^2^UCD Conway Institute of Biomolecular and Biomedical Research, University College Dublin, Dublin, Ireland

**Keywords:** biomarkers, dietary advice, dietary quality, lipids, metabolic subgroups, metabolomics, metabotypes, personalised nutrition

## Abstract

**Background:**

In a 12-week randomised controlled trial, personalised nutrition delivered using a metabotype framework improved dietary intake, metabolic health parameters and the metabolomic profile compared to population-level dietary advice. The objective of the present work was to investigate the patterns of dietary advice delivered during the intervention and the alterations in dietary intake and metabolic and metabolomic profiles to obtain further insights into the effectiveness of the metabotype framework.

**Methods:**

Forty-nine individuals were randomised into the intervention group and subsequently classified into metabotypes using four biomarkers (triacylglycerol, HDL-C, total cholesterol, glucose). These individuals received personalised dietary advice from decision tree algorithms containing metabotypes and individual characteristics. In a secondary analysis of the data, patterns of dietary advice were identified by clustering individuals according to the dietary messages received and clusters were compared for changes in dietary intake and metabolic health parameters. Correlations between changes in blood clinical chemistry and changes in metabolite levels were investigated.

**Results:**

Two clusters of individuals with distinct patterns of dietary advice were identified. Cluster 1 had the highest percentage of messages delivered to increase the intake of beans and pulses and milk and dairy products. Cluster 2 had the highest percentage of messages delivered to limit the intake of foods high in added sugar, high-fat foods and alcohol. Following the intervention, both patterns improved dietary quality assessed by the Alternate Mediterranean Diet Score and the Alternative Healthy Eating Index, nutrient intakes, blood pressure, triacylglycerol and LDL-C (*p* ≤ 0.05). Several correlations were identified between changes in total cholesterol, LDL-C, triacylglycerol, insulin and HOMA-IR and changes in metabolites levels, including mostly lipids (sphingomyelins, lysophosphatidylcholines, glycerophosphocholines and fatty acid carnitines).

**Conclusion:**

The findings indicate that the metabotype framework effectively personalises and delivers dietary advice to improve dietary quality and metabolic health.

**Clinical trial registration:**

isrctn.com, identifier ISRCTN15305840.

## 1. Introduction

Dietary factors play a major role in the development of cardiometabolic diseases (1). Among a variety of strategies for improving metabolic health, there is an increased interest in personalised nutrition (2, 3). The enthusiasm in this area stems from the evidence on the importance of interindividual variability in response to dietary factors (4, 5). Moreover, personalised approaches have achieved greater improvements in dietary intake and health outcomes compared to population-level nutrition (6).

Examining the metabolic phenotype (metabotyping) is a novel strategy for personalising healthcare (7–9). This approach consists of grouping individuals with similar metabolic profiles (metabotypes) and designing interventions tailored to the characteristics of the group (7). Metabotypes have been robustly associated with different incidences of diet-related diseases (10–13), which supports their use to identify groups that could benefit from personalised nutrition. In addition, several studies have demonstrated that metabotypes present differential associations with dietary factors and responses to dietary interventions (14–19). For example, in a German cohort the prevalence of type 2 diabetes within a high-risk metabotype [characterised by an unfavourable biomarker profile and high body mass index (BMI)] was positively associated with the intake of sugar-sweetened beverages and inversely associated with fruit intake, while the prevalence of type 2 diabetes in the metabotypes with more beneficial metabolic profiles was positively associated with the intake of total meat and processed meats (19). However, few studies investigated the use of metabotypes to deliver personalised nutrition. A dietary intervention demonstrated that the modulation of the macronutrient composition of diets prompted different cardiometabolic responses in metabotypes of tissue-specific (liver or muscle) insulin resistance (20), but dietary advice tailored for metabotypes based on extensive metabolomic and genetic profiling was not superior to generic dietary advice to improve body weight and metabolic health parameters in overweight and obese individuals (21). Although there is evidence supporting the use of metabotypes to develop dietary interventions targeted at a group level for the improvement of metabolic health and prevention of diet-related diseases, the methods applied and findings are not consistent.

Recently, we demonstrated with a 12-week randomised controlled trial (RCT) that personalised dietary advice based on metabotypes for a generally healthy population produced greater improvements in dietary quality and lipid profile (reductions in total cholesterol, LDL-C, triacylglycerol and glycerophosphocholines) compared to population-level nutrition advice (22). The framework applied comprises the classification of individuals into metabotypes using metabolic health markers and the delivery of dietary advice through a decision tree approach (23, 24), which results in a set of food or food group-related messages that are personalised for each individual. In order to obtain further insights into the mechanisms of the effectiveness of the metabotype framework, the objectives of the present paper were to investigate the patterns of dietary advice delivered during the intervention and the alterations in dietary intake and metabolic and metabolomic profiles.

## 2. Methods

The present paper reports secondary analyses carried out on data from participants that received personalised nutrition advice using a metabotype framework as part of a 12-week single-blind parallel RCT (ISRCTN15305840) (22). Throughout the study, participants attended three visits (baseline, week 4, and week 12) and received reports with dietary advice following baseline and week 4 visits. Free-living and healthy men and women aged 18 to 65 years and with a BMI ≥18.5 kg/m^2^ were eligible to participate in the study. The exclusion criteria were as follows: any metabolic disease or condition that possibly alters energy metabolism or nutritional requirements (e.g., cancer, thyroid disorders, diabetes, inflammatory bowel diseases), food allergies or intolerances, adherence to a diet prescribed for any reason, adherence to a vegan diet, concomitant participation in another clinical trial, pregnancy, lactation and planning to become pregnant within the study period. Following the baseline visit, a total of 54 participants were randomised to the personalised group to receive dietary advice based on the metabotype framework. One participant in the personalised group did not receive the allocated intervention as the 4-day food diary was not returned and four participants dropped out during the study mainly due to COVID-19-related issues. The study was approved by the ethics review board of the University College Dublin (LS-19-98-Brennan), performed in accordance with the declaration of Helsinki and all enrolled participants provided written informed consent.

In the RCT, the primary outcome was dietary quality assessed by the Alternate Mediterranean Diet Score (AMED) (25). Secondary outcomes were dietary quality assessed by the Alternative Healthy Eating Index 2010 (AHEI) (26), metabolic health biomarkers from lipid (total cholesterol, LDL-C, HDL-C, triacylglycerol) and glycaemic [glucose, insulin, homeostatic model assessment for insulin resistance (HOMA-IR)] parameters, anthropometry (weight, BMI, waist circumference, waist-hip ratio), systolic and diastolic blood pressures and blood metabolites levels using metabolomic analysis.

### 2.1. Delivery of personalised dietary advice

Participants received personalised nutrition advice based on a metabotype framework previously published (23, 24). The advice consisted of two reports containing three dietary goals each that were delivered following baseline and week 4 visits (22). Following week 4, participants were asked to follow both reports at the same time. To obtain the dietary goals, participants were classified into their metabotypes using a clustering model defined by *k*-means cluster analysis and four biomarkers of metabolic health (total cholesterol, HDL-C, triacylglycerol and glucose) (23). Subsequently, personalised dietary messages for each participant were derived from decision trees algorithms containing information on the metabotype characteristics and individual BMI, waist circumference and blood pressure (22, 24). The personalised dietary messages were then merged and expanded to compose the dietary goals (22). During the intervention, participants were supported by regular email contact.

### 2.2. Dietary, anthropometric and clinical measurements

A detailed description of data collection was previously provided (22). Dietary intake was assessed before baseline and week 12 visits using a 4-day food diary. Energy and nutrient intakes were estimated using Nutritics software (Nutritics, Ireland) and are reported as excluding the intake of supplements. Dietary quality was assessed by AMED (25) and AHEI scores (26). The AMED consists of 9 components with a total score ranging from 0 to 9 points and the AHEI consists of 11 components with a total score ranging from 0 to 110 points. For both indexes, higher values indicate a diet that aligns better with dietary recommendations.

Anthropometric and clinical data collection was performed following a 12 h overnight fast. Anthropometric measurements included weight, height, waist circumference and hip circumference. Blood pressure was measured from the reference arm using an automatic blood pressure monitor. Blood was collected by a trained phlebotomist using a lithium heparin tube, processed following a standardised procedure (centrifuged at 1800 g for 10 min at 4°C and stored in 500 μL aliquots at −80°C) and plasma was used for clinical chemistry and metabolomic analyses. Clinical chemistry markers were quantified using colourimetric (total cholesterol, LDL-C, HDL-C, triacylglycerol and glucose; Sigma-Aldrich, Germany) and ELISA (insulin; Mercodia, Sweden) assays. Quality control (Randox Health, United Kingdom) and pooled plasma samples were included in the analytical runs. HOMA-IR was calculated as [(fasting insulin μU/mL × fasting glucose mmol/L)/22.5].

### 2.3. Metabolomic analyses

Targeted metabolomic analysis was performed on plasma samples using the AbsoluteIDQ p180 kit (Biocrates, Austria). Samples were analysed in three batches and samples from an individual were prepared in the same batch with positions randomised in the assay plate. The assay plate was preset with internal standards. Ten μL of test sample (phosphate-buffered saline, calibration standard solutions, quality controls, plasma or pooled plasma) was added to capture assay plate and dried at room temperature for 30 min. Samples were then derivatised with 50 μL of solution (5% phenyl isothiocyanate in ethanol/water/pyridine, volume ratio 1/1/1), incubated at room temperature for 25 min and dried under a nitrogen stream for 60 min. Metabolites were extracted with 300 μL of 5 mM ammonium acetate in methanol by shaking the plate for 30 min and following centrifugation at 500 g for 2 min. For LC-MS/MS analysis, 150 μL of the eluate was diluted in HPLC water (150 μL) and for FIA-MS/MS analysis, 50 μL of eluate was diluted with 450 μL of methanol.

Samples were analysed on a Sciex QTRAP 6500+ mass spectrometer coupled to a Sciex ExionLC series UHPLC capability as previously published (27). A total of 20 amino acids and nine biogenic amines were identified using LC-MS/MS in positive mode. Lipids [carnitine, 12 fatty acid carnitines (C), 10 monoacylglycerophosphocholines (LPC), 33 diacylglycerophosphocholines (PC), 37 1-alkyl,2-acylglycerophosphocholines (PC-O), 14 sphingomyelins (SM)] and the sum of hexoses were identified using FIA-MS/MS in positive mode.

Amino acids and biogenic amines were quantified using isotopically labelled internal standards and seven-point calibration curves in the AB Sciex Analyst software v 1.7.2. Lipids and the sum of hexoses were measured semi-quantitatively by using 14 internal standards in MetIDQ software. MetIDQ was also used to check the quality of data through accuracy and reproducibility of QC samples (Supplementary Table S1). Only metabolites with levels above the limit of detection in >75% of samples were included in statistical analyses, which are reported in micromoles.

### 2.4. Statistical analyses

To identify patterns of dietary advice delivered through the metabotype framework, dietary advice clusters were generated using a total of 22 dietary messages that compose the metabotype framework (24). Each dietary message was considered a categorical variable and coded as 0 or 1 based on whether the participant received it or not during the intervention. The SPSS two-step cluster analysis procedure with the log-likelihood distance measures was performed to identify the clusters and the Schwarz’s Bayesian Criterion was used to automatically determine the optimal number of clusters. The normality of dietary intake data and metabolic health parameters was determined through visual inspection of histograms and Q–Q plots. Non-normal distributed variables were transformed using log10. At baseline, differences between dietary advice clusters were assessed by chi-square test (categorical variables) and independent *t*-test (continuous variables). Post-intervention (week 12), differences between dietary advice clusters were assessed by linear mixed models that included dietary advice clusters (two levels: cluster 1 vs. cluster 2), time (two levels: baseline vs. week 12) and their interaction as fixed effects and random intercepts for participants. Benjamini-Hochberg procedure was used to control multiple testing between clusters and a false discovery rate (FDR) ≤0.05 was considered significant. The changes in blood clinical chemistry and metabolite levels were calculated as the value at baseline minus the value at week 12 visit. To investigate the associations between changes in blood clinical chemistry with changes in metabolite levels, Spearman correlations and partial Spearman correlations controlled for age, sex and weight loss were applied to all participants as one group. The network graph was created in Cytoscape v 3.7.2 (28) using correlation coefficients ≤ −0.4 or ≥0.4. Statistical analyses were performed using SPSS v 27 (IBM, United States).

## 3. Results

### 3.1. Dietary advice clusters have distinct metabolic profiles

A total of 49 participants completed the intervention, however, four participants did not have blood samples available. For the present analyses, a total of 45 participants with dietary and blood data available at the completion of the intervention were included. Clustering of participants according to the dietary messages received resulted in two clusters with a silhouette measure of cohesion and separation of 0.5. Cluster 2 was characterised by the highest BMI, blood pressure, LDL-C, triacylglycerol, insulin, HOMA-IR and the lowest HDL-C and AMED and AHEI scores (Table 1). Additionally, cluster 2 had the highest percentage of participants that received dietary messages to limit the intake of foods high in added sugar, high-fat foods and alcohol and to implement low-fat cooking strategies (Table 2). By contrast, cluster 1 was characterised by a healthier metabolic profile and higher dietary quality and had the highest percentage of participants that received dietary messages to increase the intake of beans and pulses and milk and dairy products. Age, total cholesterol and glucose were similar between clusters at baseline. Furthermore, the percentages of participants that received dietary messages targeted at increasing the intake of fibre and unsaturated fat and reducing the intake of saturated fat were similar across the two clusters.

**Table 1:** Baseline characteristics of participants by dietary advice cluster.

	Dietary advice cluster 1 (*n* = 22)	Dietary advice cluster 2 (*n* = 23)	*p*-value	FDR
AMED	4.41 ± 1.68	3.22 ± 1.88	**0.030**	**0.040**
AHEI	53.1 ± 14.6	47.0 ± 14.9	0.175	0.199
Sex, male/female (*n*)	9/13	11/12	0.641	0.641
Age (years)	44.2 ± 10.9	47.5 ± 8.7	0.268	0.285
Weight (kg)^a^	64.4 ± 12.2	86.1 ± 13.2	**<0.001**	**<0.001**
BMI (kg/m^2^)^a^	22.3 ± 2.0	28.7 ± 2.4	**<0.001**	**<0.001**
Waist circumference (cm)	77 ± 8	101 ± 10	**<0.001**	**<0.001**
Waist-hip ratio	0.79 ± 0.07	0.92 ± 0.07	**<0.001**	**<0.001**
Systolic blood pressure (mmHg)^a^	106 ± 12	122 ± 15	**<0.001**	**0.001**
Diastolic blood pressure (mmHg)^a^	62 ± 6	73 ± 8	**<0.001**	**<0.001**
Total cholesterol (mmol)	4.64 ± 1.02	5.32 ± 0.96	**0.024**	**0.034**
LDL-C (mmol)	3.60 ± 0.88	4.41 ± 0.82	**0.003**	**0.005**
HDL-C (mmol)	1.63 ± 0.50	1.22 ± 0.53	**0.009**	**0.014**
Triacylglycerol (mmol)^a^	0.42 ± 0.22	0.86 ± 0.56	**0.003**	**0.005**
Glucose (mmol)	4.63 ± 0.40	4.89 ± 0.65	0.116	0.140
Insulin (mU/L)^a^	5.13 ± 1.64	11.57 ± 8.56	**<0.001**	**<0.001**
HOMA-IR^a^	1.06 ± 0.36	2.63 ± 2.42	**<0.001**	**<0.001**

Values are mean ± standard deviation. *p*-values are the comparison between clusters using chi-square for categorical variables and independent *t*-test for continuous variables. AMED, Alternate Mediterranean Diet Score; AHEI, Alternative Healthy Eating Index 2010; BMI, body mass index; FDR, false discovery rate; HOMA-IR, homeostatic model assessment for insulin resistance; HDL-C, high-density lipoprotein cholesterol; LDL-C, low-density lipoprotein cholesterol.

a Variables were log10 transformed prior to analyses. Bold values are values significant at *p* ≤0.05 or FDR ≤0.05.

**Table 2:** Percentage of participants in each dietary advice cluster that received the dietary messages from the metabotype framework, considering both reports as a total or each report separately.

	Dietary advice cluster 1 (*n* = 22)			Dietary advice cluster 2 (*n* = 23)		
	Baseline + week 4	Baseline	Week 4	Baseline + week 4	Baseline	Week 4
**Messages focused on dietary quality improvement**						
Limit the intake of foods high in added sugar	50.0	40.9	18.2	95.7	95.7	34.8
Reduce the intake of high-fat foods	13.6	13.6	0	91.3	73.9	52.2
Low-fat cooking advice	13.6	13.6	0	73.9	26.1	56.5
Eat oily fish twice a week	95.5	77.3	22.7	87.0	8.7	82.6
Have a small daily handful of seeds and nuts	95.5	77.3	22.7	87.0	8.7	82.6
Choose fibre-rich carbohydrates	95.5	54.5	77.3	100	95.7	69.6
Eat five servings of fruit and vegetables per day	95.5	59.1	50.0	95.7	47.8	52.2
Eat more dark green vegetables	86.4	27.3	77.3	78.3	26.1	56.5
Eat more beans and pulses	81.8	31.8	68.2	56.5	26.1	30.4
Choose lean meats	63.6	50.0	45.5	73.9	73.9	13.0
Choose low-fat dairy products	72.7	63.6	45.5	73.9	73.9	17.4
Limit the intake of processed foods	72.7	63.6	45.5	78.3	78.3	26.1
Eat 3 servings of dairy products per day	50.0	31.8	22.7	13.0	4.3	8.7
Limit alcohol intake	9.1	0	9.1	30.4	21.7	13.0
Choose low-salt products	0	0	0	8.7	8.7	4.3
Limit added salt	0	0	0	8.7	8.7	4.3
**Messages focused on body weight management**						
Aim for a gradual weight loss (0.5–1 kg per week)	4.5	4.5	0	100	95.7	100
Exercise for 60–90 min per day	4.5	4.5	0	100	95.7	100
Do not skip breakfast and avoid eating at night-time	4.5	4.5	0	100	95.7	100
Reduce the size of food servings	4.5	4.5	0	100	95.7	100
You have a healthy body weight: aim to keep it	100	95.5	100	4.3	4.3	0
Exercise for 30 min per day	100	95.5	100	4.3	4.3	0

### 3.2. Distinct patterns of dietary advice similarly improve dietary intake and metabolic health parameters

Following 12 weeks of intervention, the delivery of personalised dietary advice improved dietary quality assessed by the AMED and AHEI scores and several dietary intakes and metabolic health parameters in the total group (Table 3, time effect FDR ≤0.05). Although the personalisation generated different patterns of dietary advice, both dietary advice clusters 1 and 2 had increases in the AMED score which were equivalent to 15% and 10% of the total score, respectively. Changes in the metabolic health parameters and dietary intake were mostly similar between clusters (Table 3, cluster x time interaction FDR ≥0.05). Energy intake was greater decreased in cluster 2 (Δ cluster 1 = −101 ± 444 kcal vs. Δ cluster 2 = −423 ± 364 kcal, *p* = 0.011) and folate intake was increased in cluster 1 while decreased in cluster 2 (Δ cluster 1 = 54 ± 103 μg vs. Δ cluster 2 = −29 ± 116 μg, *p* = 0.018); however, they did not reach significance following correction for multiple testing.

**Table 3:** Impact of personalised nutrition advice on dietary intake and metabolic health parameters by dietary advice cluster.

	Dietary advice cluster 1 (*n* = 22)		Dietary advice cluster 2 (*n* = 23)		*p*-value (C)	*p*-value (T)	*p*-value (C × T)	FDR (C)	FDR (T)	FDR (C × T)
	Baseline	Week 12	Baseline	Week 12						
**Dietary intake**										
AMED	4.41 ± 1.68	5.82 ± 1.68	3.22 ± 1.88	4.13 ± 1.52	**<0.001**	**0.002**	0.370	**0.004**	**0.001**	0.635
AHEI	53.1 ± 14.6	61.7 ± 8.6	47.0 ± 14.9	51.5 ± 11.4	**0.015**	**0.002**	0.289	**0.031**	**0.005**	0.592
Energy (kcal)	2080 ± 500	1979 ± 540	2,236 ± 557	1813 ± 482	0.970	**<0.001**	**0.011**	0.982	**0.001**	0.250
Carbohydrate (% E)	41.1 ± 9.8	38.8 ± 10.6	40.6 ± 6.0	39.2 ± 5.7	0.982	0.061	0.595	0.982	0.095	0.694
Fat (% E)	39.8 ± 8.5	39.0 ± 7.1	39.9 ± 5.1	40.7 ± 5.8	0.610	0.998	0.420	0.657	0.998	0.635
Protein (% E)^a^	15.6 ± 3.8	18.5 ± 4.7	16.4 ± 3.6	18.5 ± 3.5	0.587	**<0.001**	0.515	0.657	**0.001**	0.668
Free sugars (% E)	8.2 ± 4.2	5.5 ± 4.1	9.0 ± 4.2	7.7 ± 3.8	0.172	**<0.001**	0.172	0.255	**0.003**	0.592
Saturated fat (% E)	14.9 ± 4.4	12.2 ± 2.5	15.1 ± 3.1	13.5 ± 2.8	0.373	**<0.001**	0.345	0.487	**0.001**	0.635
Polyunsaturated fat (% E)^a^	6.5 ± 1.7	7.7 ± 2.4	6.1 ± 1.6	7.1 ± 2.9	0.238	**0.043**	0.654	0.333	0.080	0.719
Monounsaturated fat (% E)^a^	14.8 ± 3.7	15.3 ± 4.0	14.5 ± 2.6	16.3 ± 3.8	0.578	0.068	0.296	0.657	0.100	0.592
Fibre (g/1000 kcal)^a^	10.9 ± 2.7	13.8 ± 3.6	10.2 ± 2.8	11.6 ± 2.8	0.098	**<0.001**	0.196	0.172	**0.001**	0.592
Sodium (mg)^a^	2,435 ± 645	2,164 ± 923	2,871 ± 747	2,131 ± 816	0.382	**<0.001**	0.098	0.487	**<0.001**	0.592
Calcium (mg)^a^	997 ± 315	892 ± 319	995 ± 402	806 ± 329	0.470	**0.009**	0.525	0.572	**0.022**	0.668
Folate (μg)^a^	307 ± 93	361 ± 122	309 ± 115	280 ± 100	0.126	0.661	**0.018**	0.208	0.736	0.250
**Metabolic health parameters**										
Weight (kg)^a^	64.4 ± 12.2	64.0 ± 12.4	86.1 ± 13.2	85.0 ± 13.7	**<0.001**	0.056	0.454	**<0.001**	0.091	0.635
BMI (kg/m^2^)^a^	22.3 ± 2.0	22.2 ± 2.2	28.7 ± 2.4	28.3 ± 2.7	**<0.001**	0.056	0.454	**<0.001**	0.091	0.635
Waist circumference (cm)	77 ± 8	77 ± 9	101 ± 10	99 ± 11	**<0.001**	**0.029**	0.287	**<0.001**	0.059	0.592
Waist-hip ratio	0.79 ± 0.07	0.79 ± 0.07	0.92 ± 0.07	0.92 ± 0.09	**<0.001**	0.261	0.668	**<0.001**	0.331	0.719
Systolic blood pressure (mmHg)^a^	106 ± 12	106 ± 14	122 ± 15	118 ± 12	**<0.001**	0.213	0.214	**0.001**	0.283	0.592
Diastolic blood pressure (mmHg)^a^	62 ± 6	62 ± 7	73 ± 8	70 ± 7	**<0.001**	**0.017**	0.238	**<0.001**	**0.037**	0.592
Total cholesterol (mmol)	4.64 ± 1.02	4.69 ± 0.99	5.32 ± 0.96	5.01 ± 0.96	0.067	0.272	0.119	0.125	0.331	0.592
LDL-C (mmol)	3.60 ± 0.88	3.22 ± 0.77	4.41 ± 0.82	4.26 ± 0.84	**<0.001**	**0.011**	0.256	**0.001**	**0.026**	0.592
HDL-C (mmol)	1.63 ± 0.50	1.79 ± 0.58	1.22 ± 0.53	1.22 ± 0.51	**0.002**	0.160	0.150	**0.004**	0.225	0.592
Triacylglycerol (mmol)^a^	0.42 ± 0.22	0.31 ± 0.19	0.86 ± 0.56	0.57 ± 0.33	**<0.001**	**0.001**	0.915	**0.001**	**0.005**	0.949
Glucose (mmol)	4.63 ± 0.40	4.69 ± 0.36	4.89 ± 0.65	4.76 ± 0.41	0.173	0.609	0.227	0.255	0.711	0.592
Insulin (mU/L)^a^	5.13 ± 1.64	5.57 ± 2.71	11.57 ± 8.56	10.66 ± 6.24	**<0.001**	0.712	0.580	**<0.001**	0.738	0.694
HOMA-IR^a^	1.06 ± 0.36	1.17 ± 0.60	2.63 ± 2.42	2.28 ± 1.41	**<0.001**	0.683	0.449	**<0.001**	0.736	0.635

Values are mean ± standard deviation. *p*-values are the comparison between clusters using linear mixed models: C, cluster effect; T, time effect; C × T, cluster by time interaction; AMED, Alternate Mediterranean Diet Score; AHEI, Alternative Healthy Eating Index 2010; BMI, body mass index; FDR, false discovery rate; HOMA-IR, homeostatic model assessment for insulin resistance; HDL-C, high-density lipoprotein cholesterol; LDL-C, low-density lipoprotein cholesterol; % E, percentage of total energy intake.

a Variables were log10 transformed prior to analyses. Bold values are values significant at *p* ≤0.05 or FDR ≤0.05.

### 3.3. Changes in blood clinical chemistry correlate with changes in circulating metabolites

**Figure 1:**
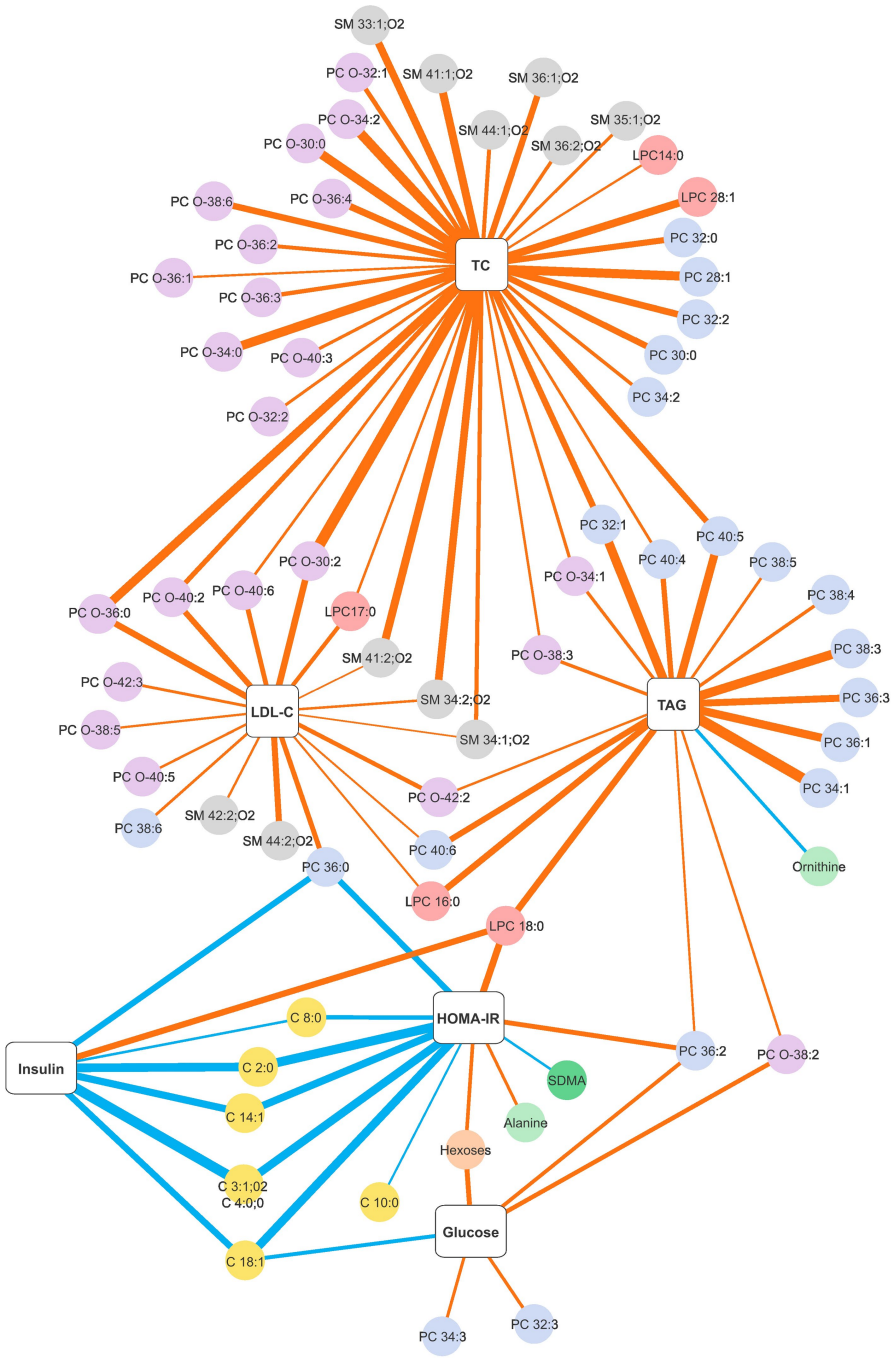
Network graph of significant Spearman correlation coefficients between changes in the blood clinical chemistry and changes in plasma metabolite levels obtained with a personalised dietary advice intervention. The thickness of the strokes denotes the magnitude of the correlation, blue strokes denote negative correlations and orange strokes denote positive correlations. Only correlation coefficients ≤ −0.4 or ≥0.4 are presented. The strongest negative correlation is *r* = −0.607 and the strongest positive correlation is *r* = 0.631. The complete list of significant correlations is presented in Supplementary Figure S1. C, fatty acid carnitines; HOMA-IR, homeostatic model assessment for insulin resistance; LDL-C, low-density lipoprotein cholesterol; LPC, monoglycerophosphocholines; PC-O, 1-alkyl,2-acylglycerophosphocholines; PC, diacylglycerophosphocholines; SM, sphingomyelins; TAG, triacylglycerol; TC, total cholesterol.

Since the dietary intervention resulted in similar improvements in metabolic health parameters for both dietary advice clusters, we investigated the correlations between the changes in the concentration of blood clinical chemistry and the changes in metabolite levels for the intervention group as a whole. Changes in a number of clinical biomarkers were significantly correlated with metabolite changes with the highest number observed for total cholesterol (63 significant correlations). Significant correlation coefficients ≤−0.4 or ≥0.4 are presented in Figure 1 and the totality of significant correlations is presented in Supplementary Figure S1. Changes in total cholesterol and LDL-C were positively correlated with changes in sphingomyelins and glycerophosphocholines, mostly in the subclass PC-O. The strongest correlations with changes in total cholesterol were with SM 33:1;O2, SM 34:2;O2, SM 41:1;O2, SM 41:2;O2 and glycerophosphocholines of shorter chain lengths (LPC 28:1, PC 28:1, PC 32:2, PC O-30:0, PC O-30:2, PC O-34:0, PC O-34:2, PC O-36:0 and PC O-36:4). Although changes in LDL-C followed a similar pattern of positive correlations with the abovementioned lipid classes, they were less numerous and the coefficients were weaker. On the contrary, changes in HDL-C were not strongly correlated with metabolite changes. Changes in triacylglycerol were mostly positively correlated with changes in glycerophosphocholines in the subclass PC, with the strongest coefficients for the monounsaturated PC 32:1, PC 34:1, PC 36:1, and for the polyunsaturated PC 38:3 and PC 40:5. A negative significant correlation of triacylglycerol was observed with the amino acid ornithine. For changes in glucose, a few positive correlations were observed with the subclasses PC and PC-O. However, more interestingly, several inverse correlations were observed between changes in insulin and HOMA-IR with changes in fatty acid carnitines and PC 36:0. Additionally, changes in insulin presented a positive correlation with LPC 18:0 while changes in HOMA-IR presented positive correlations with LPC 18:0, PC 36:2 and alanine and a negative correlation with symmetric dimethylarginine (SDMA).

Analyses of anthropometric and demographic parameters revealed significant positive correlations between weight loss during the intervention and changes in glucose, insulin and HOMA-IR (0.354 ≤ *r* ≥ 0.404) and a negative correlation between age and change in HDL-C (*r* = −0.303, Supplementary Table S2). However, partial correlations between changes in blood clinical chemistry and metabolites controlled for weight loss, age and sex did not affect substantially the number, strength and direction of correlations (Supplementary Figure S2).

## 4. Discussion

Analyses of the personalised dietary messages delivered through the metabotype framework during the intervention identified two clusters of participants (dietary advice clusters) with distinct patterns of dietary advice. Both patterns of dietary advice similarly improved dietary quality assessed by the AMED and AHEI scores, nutrient intakes and metabolic health parameters. In addition, several correlations were identified between changes in blood clinical chemistry and changes in metabolite levels, which included mostly lipids (sphingomyelins, glycerophosphocholines and fatty acid carnitines). These findings indicate that the metabotype framework effectively personalises and delivers dietary advice to improve dietary quality and metabolic health parameters. Importantly, the different patterns of advice resulted in similar improvements in overall dietary quality and metabolic health highlighting the power of personalising based on the metabotype.

Personalised nutrition is built on the premise that dietary advice tailored to an individual will be more effective than one-size-fits-all approaches to improve dietary intake and metabolic health (2, 3, 29). The process includes the identification of individual characteristics that, if targeted, could induce the desired changes in dietary behaviour and consequently in health outcomes. However, the success of an approach will also depend on its ability to translate this information into practical and feasible advice. This is important as dietary changes require individuals to make commitments with daily choices and those perceived as personally relevant are more likely to be remembered and assumed to increase motivation and compliance (2, 29–31). As a tool for delivering dietary advice to healthy individuals, the metabotype framework guides the prioritisation of dietary messages that align with population-based recommendations by harnessing the individual metabolic profile (3). This is evident in the delivery of messages targeted at increasing the intake of unsaturated fat and fibre. In the RCT, participants in the control group received population-level nutrition advice containing the same six dietary goals based on the national dietary guidelines but in random order (22). Although messages with potential benefits on the intake of fibre and saturated fat were present in four and five dietary goals respectively, dietary advice delivered using the metabotype framework resulted in greater improvements in the intake of these nutrients. In the present analysis, two different patterns of dietary advice resulted in similar improvements in AMED and AHEI scores, which are effective indicators of dietary quality (32–34), and in several nutrient intakes reflecting the focus of the optimisation of the metabotype framework to tailor dietary advice (24). Dietary messages targeted at reducing sodium intake were delivered to a few participants but the reduction in the intake is possibly a consequence of the improvement of the overall dietary quality (35). These results reinforce the ability of the metabotype framework to improve dietary intake through the personalisation of dietary advice.

Several personalised approaches using lifestyle, phenotypic or genotypic information were successful at tailoring dietary advice that promoted dietary improvements (36–40). However, the use of metabotypes for this purpose is emerging and the available studies present critical differences in their approaches (20–22). The 12-week RCT PERSON study investigated the use of diets tailored for metabotypes of muscle or liver insulin resistance to improve cardiometabolic health (20). The metabotypes defined in the study considered the groups as having unique profiles that would benefit from distinct diets and individuals with heterogeneous profiles were not included (41). Although the primary outcome (disposition index) was not impacted by the intervention, secondary outcomes (insulin sensitivity, glucose homeostasis, triacylglycerol and C-reactive protein) were improved among individuals who followed the considered suboptimal diets. These results evidence the opportunity to use diets modulated based on metabotypes to achieve meaningful clinical improvements in cardiometabolic health but the complexity of delivering personalised nutrition. The 10-week RCT PREVENTOMICS focused on improving body weight and composition of overweight and obese individuals by scoring them into five metabolic processes/metabotypes (oxidative stress, inflammation, carbohydrate metabolism, lipid metabolism and gut microbiota metabolism) using an algorithm with 51 urine and blood markers and 35 SNPs to score (21). Following a personalised dietary plan based on their metabotype did not improve the outcomes further compared to generic advice. However, the individuals followed a diet with the characteristics solely of the metabolic process where they scored the highest while metabolic processes with lower scores but potentially relevant were not be addressed in the dietary plan (42). Compared to the abovementioned studies, the metabotype framework used here employs a more flexible approach to define the metabotypes and tailor dietary advice. For example, it has two metabotypes characterised by high average total cholesterol but their other characteristics are distinct enough so that the dietary advice can be tailored. In addition, the metabotype framework includes variables in the decision trees that allow further personalisation of dietary advice. Metabotypes with similarities in the metabolic profile are common, especially in diabetes research (10, 11, 43, 44). Characteristics that are shared among metabotypes reflect the heterogeneity of the human phenotype which must be considered when tailoring dietary advice and may have contributed to the effectiveness of the metabotype framework.

In addition to dietary quality and nutrient intakes, tailoring dietary advice using the metabotype framework improved metabolic health parameters with both patterns of dietary advice. Personalised nutrition approaches have mostly reported improvements in dietary intake (6, 36, 45), while one approach applied to three different populations reported improvements only in metabolic health parameters (5, 46, 47). Studies with positive effects in both dimensions are scarce. A 12-week RCT that applied the Food4Me decision trees adapted to overweight and obese Chinese adults (*n* = 318) found that the personalised group significantly increased dietary quality (China dietary guidelines index) and physical activity levels and reduced anthropometric parameters (e.g., BMI, body fat percentage, waist circumference) and blood biomarkers concentrations (e.g., blood lipids, uric acid, homocysteine) compared to generic advice (48). Personalised messages to lose weight, increase fibre intake and take multivitamin/mineral supplements were identified as the major contributors to reducing BMI and improving the lipid profile. The concomitant improvement in dietary quality and metabolic outcomes is solid evidence to support personalised approaches to deliver dietary advice. With improvements in dietary quality and lipid profile, the metabotype framework emerges as a tool for addressing well-established risk factors for cardiometabolic diseases and consequently their prevention.

Metabolomics produces high-resolution and comprehensive biological signatures that have the potential to provide a broader understanding of metabolism (49, 50). Previous prospective metabolomic studies have established that several metabolites are differentially linked to the risk of cardiometabolic diseases including altered metabolism of amino acids, acylcarnitines, sphingolipids and phospholipids (51–53). However, their changes in relation to the classical blood markers are less frequently reported, especially for dietary interventions. In our study, changes in total cholesterol and LDL-C were mostly positively correlated with changes in sphingomyelins and glycerophosphocholines with shorter chain lengths. Similar associations were demonstrated in an intervention study during periods of weight loss and weight maintenance indicating that the associations were independent of weight decrease (54). These associations are expected as sphingomyelins are sphingolipids found predominantly in circulating LDL-C, while glycerophosphocholines are the most abundant lipid subclass in LDL-C (55). However, most of the glycerophosphocholines associated with changes in total cholesterol and LDL-C had alkyl-acyl residues (PC-O) while the diacyl residues (PC) were mostly associated with changes in triacylglycerol. PC-O are phospholipids characterised by a hydrocarbon chain with an ether bond located at the sn-1 position of the glycerol-backbone and often include plasmalogens, which have a vinyl-ether linkage (56). Plasmalogens are implicated in cholesterol transport and one possibility is that their increased concentration may indicate increased protective activity against oxidative stress. Findings from three Dutch population-based cohorts with 5,337 individuals reported positive correlations between concentrations of LDL-C and levels of several glycerophosphocholines with diacyl residues (PC 36:2, PC 36:3, PC 36:4, PC 38:5, PC 38:6) while concentrations of triacylglycerol were positively correlated with levels of several glycerophosphocholines with alky-acyl residues (PC O-34:1, PC O-36:1, PC O-36:2, PC O-36:3, PC O-38:3, PC O-40:4, PC O-40:6) (57). In addition, the strongest correlations with changes in triacylglycerol were observed with changes in monounsaturated diacyl residues. Furthermore, we observed inverse associations between changes in several fatty acid carnitines and changes in insulin levels and HOMA-IR. Population-based studies have associated increased levels of fatty acid carnitines with incident type 2 diabetes (58, 59), especially those with long carbon chains (60). However, interventions with weight loss and reduced fructose intake demonstrated a general increase in fatty acid carnitines levels (61, 62). In the EPIC cohort, these levels were influenced by intrinsic and lifestyle factors in different directions which reflects the complexity of the topic (63). Changes in blood biochemistry were also correlated with changes in the levels of amino acids and derivates. Changes in HOMA-IR were positively correlated with changes in alanine and inversely correlated with changes in SDMA. Alanine is one of the most abundant amino acids in the circulation and higher levels have been robustly associated with impaired insulin secretion and incident type 2 diabetes (51, 64). On the contrary, the status of SDMA in insulin resistance is controversial. Elevated SDMA levels have been associated with several clinical conditions, including type 2 diabetes (65), but inverse concurrent changes in SDMA and HOMA-IR were also reported (66, 67). The associations between changes in blood clinical chemistry and metabolite levels observed with the personalised dietary advice delivery by the metabotype framework shed light on the physiological impact of the improvement of dietary quality.

A limitation of the present study is that the sample size was calculated to detect a difference in the AMED score between personalised and control groups in the primary analysis of the 12-week RCT and thus a reduced statistical power to detect difference between dietary advice clusters is expected. Additional studies with larger sample sizes could confirm if the magnitude of changes is similar between clusters. However, both presented improvements in dietary quality and metabolic health parameters which support the metabotype framework as an effective tool to tailor and deliver dietary advice. In addition, we used a targeted approach to assess the metabolomic profile of the participants and a method with a wider selection of biomarkers could provide further insights into the associations between changes in blood biochemistry and metabolites. Strengths of the study include dietary data captured through a detailed 4-day food diary in conjunction with blood clinical chemistry and extensive metabolomic data, which provides a comprehensive analysis of the effects of personalising dietary advice through the metabotype framework. In addition, both clusters of dietary advice had a wide age range but were significantly different in body weight and AMED scores at baseline. With both clusters highly benefiting from the personalisation of the advice (15 and 10% increase in AMED score for clusters 1 and 2, respectively), this suggests broad applicability of the metabotype framework to the general population.

In conclusion, the metabotype framework is an effective tool to personalise and deliver dietary advice. Concomitant improvements in dietary quality and metabolic health parameters with different patterns of dietary advice highlight the power of personalising nutrition based on metabotypes. Future development should focus on extending this approach to different population groups and evaluating its acceptance among healthcare professionals.

## Data availability statement

The data presented in the study are deposited in the MetaboLights repository (https://www.ebi.ac.uk/metabolights/), accession number MTBLS5310.

## Ethics statement

The studies involving humans were approved by University College Dublin Research Ethics Committee. The studies were conducted in accordance with the local legislation and institutional requirements. The participants provided their written informed consent to participate in this study.

## Author contributions

EH: Conceptualization, Data curation, Formal analysis, Funding acquisition, Investigation, Writing – original draft, Writing – review & editing. LB: Conceptualization, Funding acquisition, Methodology, Project administration, Resources, Supervision, Writing – review & editing.
