# Supplementary material for: Distinct patterns of personalised dietary advice delivered by a metabotype framework similarly improve dietary quality and metabolic health parameters: secondary analysis of a randomised controlled trial
Source: Front Nutr. 2023 Nov 15;10:1282741. doi: 10.3389/fnut.2023.1282741 (PMC10684740; doi:10.3389/fnut.2023.1282741)
Supplement: Supplementary file 1 [file Data_Sheet_1.PDF]

Distinct patterns of personalised dietary advice delivered by a metabotype framework similarly improve dietary quality and metabolic health parameters: secondary analysis of a randomised controlled trial

Elaine Hillesheim[1,2*], Lorraine Brennan[1,2]

[1] UCD School of Agriculture and Food Science, Institute of Food and Health, University College Dublin, Dublin, Ireland

[2] UCD Conway Institute of Biomolecular and Biomedical Research, University College Dublin, Dublin, Ireland

*** Correspondence:**
Corresponding Author
elaine.hillesheim@ucd.ie

Supplementary Table 1. Coefficient of variation inter-batches for metabolites in the quality control sample.

Metabolite	CV (%)
Alanine	1.0
Arginine	3.2
Asparagine	2.6
Citrulline	1.4
Glutamine	0.9
Glutamate	1.0
Glycine	2.2
Histidine	2.2
Isoleucine	0.9
Leucine	2.2
Lysine	5.1
Methionine	2.9
Ornithine	2.4
Phenylalanine	0.9
Proline	3.0
Serine	2.4
Threonine	0.9
Tryptophan	3.3
Tyrosine	2.3
Valine	4.5
ADMA	0.7
α-AAA	3.5
Creatinine	0.5
Kynurenine	0.3
Putrescine	0.4
Sarcosine	1.0
SDMA	6.3
t4-OH-Pro	0.6
Taurine	1.4
Hexoses	2.0
Carnitine	2.5
C 2:0	2.6
C 3:0	3.7
C 4:0	4.3
C 3:1;O2 (C 4:0;0)	1.9
C 6:0 (C 4:2;O2)	0.8
C 8:0	1.4
C 10:0	0.8
C 12:0	0.3
C 14:1	34.6
C 16:0 (C 14:2;O2)	1.2
C 18:0	2.3
C 18:1	0.7
LPC 14:0	2.2
LPC 16:0	1.0
LPC 16:1	1.2
LPC 17:0	0.8
LPC 18:0	1.8
LPC 18:1	1.4
LPC 18:2	0.6
LPC 20:3	1.6
LPC 20:4	1.6
LPC 28:1	18.6
SM 33:1;O2	2.3
SM 34:1;O2	0.3
SM 34:2;O2	2.6
SM 35:1;O2	1.0
SM 36:1;O2	2.9
SM 36:2;O2	1.8
SM 38:3;O2	2.8
SM 41:1;O2	3.1
SM 41:2;O2	3.2
SM 42:1;O2	1.0
SM 42:2;O2	2.6
SM 43:1;O2	3.2
SM 44:1;O2	1.3
SM 44:2;O2	1.1

Metabolite	CV (%)
PC 28:1	0.9
PC 30:0	13.3
PC 32:0	1.3
PC 32:1	2.3
PC 32:2	0.3
PC 32:3	1.0
PC 34:1	0.8
PC 34:2	1.6
PC 34:3	1.2
PC 34:4	1.4
PC 36:0	1.6
PC 36:1	1.0
PC 36:2	0.9
PC 36:3	1.9
PC 36:4	0.4
PC 36:5	0.8
PC 36:6	0.9
PC 38:0	0.6
PC 38:1	17.4
PC 38:3	1.7
PC 38:4	0.0
PC 38:5	0.7
PC 38:6	0.6
PC 40:2	3.1
PC 40:3	1.4
PC 40:4	1.7
PC 40:5	1.1
PC 40:6	0.3
PC 42:0	2.2
PC 42:1	0.7
PC 42:2	2.0
PC 42:4	0.8
PC 42:5	1.5
PC O-30:0	2.2
PC O-30:1	6.6
PC O-30:2	5.4
PC O-32:1	1.2
PC O-32:2	1.2
PC O-34:0	0.9
PC O-34:1	1.3
PC O-34:2	0.7
PC O-34:3	1.8
PC O-36:0	0.7
PC O-36:1	1.1
PC O-36:2	1.9
PC O-36:3	1.0
PC O-36:4	0.1
PC O-36:5	1.1
PC O-38:0	2.7
PC O-38:1	1.3
PC O-38:2	1.8
PC O-38:3	0.9
PC O-38:4	1.1
PC O-38:5	1.4
PC O-38:6	1.5
PC O-40:1	2.0
PC O-40:2	1.3
PC O-40:3	0.3
PC O-40:4	1.3
PC O-40:5	1.1
PC O-40:6	1.4
PC O-42:0	19.4
PC O-42:2	2.4
PC O-42:3	1.7
PC O-42:4	0.4
PC O-42:5	1.4
PC O-44:3	2.6
PC O-44:4	2.5
PC O-44:5	1.3
PC O-44:6	2.0

Supplementary Table 2. Spearman correlation coefficients between changes in blood clinical chemistry, weight loss and demographic parameters.

	**Δ TC**	**Δ LDL-C**	**Δ HDL-C**	**Δ TAG**	**Δ Glucose**	**Δ Insulin**	**Δ HOMA-IR**	**Weight loss**	**Age**
**Δ LDL-C**	0.275								
**Δ HDL-C**	0.294*	0.032							
**Δ TAG**	0.246	0.250	0.090						
**Δ Glucose**	0.168	0.033	0.074	0.274					
**Δ Insulin**	0.113	-0.159	0.059	0.477 **	0.365 *				
**Δ HOMA-IR**	0.113	-0.162	0.065	0.479 **	0.546 **	0.965 **			
**Weight loss**	0.233	-0.008	0.139	0.273	0.354 *	0.367 *	0.404 **		
**Age**	-0.153	-0.255	-0.303 *	0.126	0.078	-0.019	0.002	0.007	
**Sex**	0.014	-0.062	-0.103	0.062	-0.003	0.141	0.117	-0.052	-0.052

* p ≤0.05; ** p ≤0.01. HDL-C, high-density lipoprotein cholesterol; HOMA-IR, homeostatic model assessment for insulin resistance; LDL-C, low-density lipoprotein cholesterol; TAG, triacylglycerol; TC, total cholesterol.

	TC	LDL-C	HDL-C	TAG	Glucose	Insulin	HOMA
Alanine						0.397	0.426
Arginine			-0.309	-0.393			
Asparagine							
Citrulline				-0.364			
Glutamine							
Glutamate				0.344	0.376		
Glycine							
Histidine							
Isoleucine							
Leucine							
Lysine							
Methionine							
Ornithine				-0.431			
Phenylalanine							
Proline		-0.314			0.314	0.362	0.386
Serine				-0.382			
Threonine							
Tryptophan							
Tyrosine							
Valine							
ADMA							
α-AAA							
Creatinine							
Kynurenine							
Putrescine							
Sarcosine		-0.370					
SDMA					-0.399	-0.364	-0.416
t4-OH-Pro							
Taurine							
Hexoses					0.464	0.366	0.439
Carnitine							
C 2:0					-0.369	-0.568	-0.579
C 3:0							
C 4:0							
C 3:1;O2 (C 4:0;0)				-0.360		-0.607	-0.550
C 6:0 (C 4:2;O2)						-0.312	-0.343
C 8:0				-0.326		-0.417	-0.454
C 10:0				-0.331		-0.352	-0.409
C 12:0						-0.364	-0.395
C 14:1						-0.523	-0.530
C 16:0 (C 14:2;O2)						-0.308	-0.335
C 18:0	0.325						
C 18:1					-0.444	-0.500	-0.557
LPC 14:0	0.412			0.297	0.366		0.322
LPC 16:0	0.359	0.408		0.497	0.319	0.355	0.350
LPC 16:1	0.330			0.399		0.395	0.394
LPC 17:0	0.416	0.447		0.346	0.356		
LPC 18:0		0.375		0.502	0.373	0.494	0.511
LPC 18:1							
LPC 18:2							
LPC 20:3	0.325			0.340		0.331	0.322
LPC 20:4							
LPC 28:1	0.538	0.395		0.323			
SM 33:1;O2	0.530	0.358					
SM 34:1;O2	0.449	0.401					
SM 34:2;O2	0.536	0.417					
SM 35:1;O2	0.426	0.344					
SM 36:1;O2	0.496	0.386					
SM 36:2;O2	0.435	0.357					
SM 38:3;O2	0.321						
SM 41:1;O2	0.545						
SM 41:2;O2	0.545	0.400					
SM 42:1;O2	0.345						
SM 42:2;O2		0.413				-0.309	-0.360
SM 43:1;O2							
SM 44:1;O2	0.446						
SM 44:2;O2		0.490				-0.335	-0.355

	TC	LDL-C	HDL-C	TAG	Glucose	Insulin	HOMA
PC 28:1	0.579						
PC 30:0	0.495			0.364	0.334		
PC 32:0	0.491	0.341		0.391	0.300		
PC 32:1	0.494		0.323	0.573	0.306		0.310
PC 32:2	0.508			0.340	0.372	0.325	0.356
PC 32:3	0.387				0.430		
PC 34:1	0.384			0.621			
PC 34:2	0.435			0.312	0.374		
PC 34:3	0.345			0.388	0.427	0.302	0.376
PC 34:4	0.317			0.318			
PC 36:0	0.332	0.464				-0.488	-0.487
PC 36:1				0.552	0.305	0.303	0.390
PC 36:2	0.318			0.412	0.440	0.370	0.462
PC 36:3				0.517	0.373	0.309	0.380
PC 36:4	0.384			0.337			
PC 36:5							
PC 36:6	0.384						
PC 38:0	0.331	0.374					
PC 38:1							
PC 38:3	0.380			0.582			0.320
PC 38:4				0.432			
PC 38:5	0.384	0.375		0.421			
PC 38:6	0.352	0.420		0.346			
PC 40:2							
PC 40:3						0.327	0.333
PC 40:4	0.422			0.471			
PC 40:5	0.484	0.300		0.550	0.305		0.319
PC 40:6	0.369	0.406		0.484			
PC 42:0	0.350						
PC 42:1							
PC 42:2							
PC 42:4							
PC 42:5	0.329	0.330					
PC O-30:0	0.580		0.308				
PC O-30:1							
PC O-30:2	0.631	0.503					
PC O-32:1	0.457	0.353	0.338				
PC O-32:2	0.424	0.322					
PC O-34:0	0.578	0.382	0.373	0.399	0.328		
PC O-34:1	0.428			0.429			
PC O-34:2	0.594		0.383				
PC O-34:3							
PC O-36:0	0.563	0.495		0.307			
PC O-36:1	0.413	0.298	0.392	0.379			
PC O-36:2	0.444				0.389		
PC O-36:3	0.453		0.303				
PC O-36:4	0.513		0.306				
PC O-36:5	0.348	0.319					
PC O-38:0				0.318			
PC O-38:1							
PC O-38:2	0.358			0.415	0.453	0.307	0.369
PC O-38:3	0.418	0.317		0.433			
PC O-38:4	0.332	0.301	0.383				
PC O-38:5		0.413					
PC O-38:6	0.500	0.389					
PC O-40:1					0.303	0.327	0.356
PC O-40:2	0.463	0.479					
PC O-40:3	0.429	0.375					
PC O-40:4		0.302					
PC O-40:5		0.416					
PC O-40:6	0.419	0.461					
PC O-42:0							
PC O-42:2	0.345	0.452		0.414			
PC O-42:3		0.423		0.341			
PC O-42:4		0.371					
PC O-42:5							
PC O-44:3		0.377					
PC O-44:4	0.326		0.352				
PC O-44:5							
PC O-44:6							

-0.7 0.7

Supplementary Figure 1. Heatmap of Spearman correlation coefficients between changes in the blood clinical chemistry and metabolite levels obtained with a personalised dietary advice intervention. Coefficients are presented for significant correlations (p ≤0.05).

	TC	LDL-C	HDL-C	TAG	Glucose	Insulin	HOMA
Alanine							0.311
Arginine			-0.328	-0.327			
Asparagine							
Citrulline		-0.331		-0.381			
Glutamine							
Glutamate		0.390		0.323	0.372		
Glycine							
Histidine							
Isoleucine							
Leucine							
Lysine							
Methionine							
Ornithine				-0.435			
Phenylalanine							
Proline							0.310
Serine				-0.334			
Threonine							
Tryptophan							
Tyrosine							
Valine							
ADMA							
α-AAA							
Creatinine							
Kynurenine							
Putrescine							
Sarcosine		-0.424					
SDMA					-0.376	-0.359	-0.413
t4-OH-Pro				-0.369			
Taurine							
Hexoses					0.475	0.355	0.442
Carnitine							
C 2:0						-0.550	-0.555
C 3:0							
C 4:0							
C 3:1;O2 (C 4:0;O)				-0.322		-0.594	-0.526
C 6:0 (C 4:2;O2)							
C 8:0						-0.383	-0.425
C 10:0						-0.319	-0.382
C 12:0						-0.378	-0.412
C 14:1						-0.543	-0.552
C 16:0 (C 14:2;O2)						-0.309	-0.340
C 18:0	0.352						
C 18:1					-0.404	-0.459	-0.522
LPC 14:0	0.372						
LPC 16:0		0.400		0.506			
LPC 16:1				0.451		0.365	0.372
LPC 17:0	0.381	0.435		0.366	0.366		
LPC 18:0		0.382		0.484	0.313	0.447	0.462
LPC 18:1							
LPC 18:2							
LPC 20:3							
LPC 20:4							
LPC 28:1	0.524	0.392		0.315			
SM 33:1;O2	0.523	0.337					
SM 34:1;O2	0.443	0.398					
SM 34:2;O2	0.525	0.416					
SM 35:1;O2	0.439	0.357					
SM 36:1;O2	0.484	0.380					
SM 36:2;O2	0.451	0.348					
SM 38:3;O2	0.383						
SM 41:1;O2	0.531						
SM 41:2;O2	0.525	0.380					
SM 42:1;O2	0.315						
SM 42:2;O2		0.412					-0.348
SM 43:1;O2							
SM 44:1;O2	0.463						
SM 44:2;O2		0.475				-0.365	-0.393

	TC	LDL-C	HDL-C	TAG	Glucose	Insulin	HOMA
PC 28:1	0.570						
PC 30:0	0.489						
PC 32:0	0.465	0.374		0.345			
PC 32:1	0.452			0.545			
PC 32:2	0.478						
PC 32:3	0.355				0.376		
PC 34:1	0.336			0.614			
PC 34:2	0.411				0.346		
PC 34:3				0.345	0.360		
PC 34:4							
PC 36:0	0.366	0.422				-0.495	-0.485
PC 36:1				0.538			0.345
PC 36:2				0.378	0.392		0.403
PC 36:3				0.499	0.319		0.311
PC 36:4	0.353			0.333			
PC 36:5							
PC 36:6							
PC 38:0	0.338	0.343					
PC 38:1							
PC 38:3	0.332			0.559			
PC 38:4				0.473			
PC 38:5		0.352		0.442			
PC 38:6		0.390		0.366			
PC 40:2							
PC 40:3						0.314	0.324
PC 40:4	0.394			0.456			
PC 40:5	0.424			0.546			
PC 40:6		0.427		0.457			
PC 42:0	0.322						
PC 42:1							
PC 42:2							
PC 42:4							
PC 42:5		0.329					
PC O-30:0							
PC O-30:1	0.595	0.331	0.342				
PC O-30:2	0.636	0.487		0.327			
PC O-32:1	0.456	0.315					
PC O-32:2	0.444						
PC O-34:0	0.578	0.413	0.385	0.374			
PC O-34:1	0.423			0.443			
PC O-34:2	0.584		0.323				
PC O-34:3							
PC O-36:0	0.538	0.517					
PC O-36:1	0.415		0.377	0.422			
PC O-36:2	0.444				0.419		
PC O-36:3	0.422						
PC O-36:4	0.533						
PC O-36:5	0.370						
PC O-38:0				0.324			
PC O-38:1							
PC O-38:2	0.316			0.343	0.364		
PC O-38:3	0.393			0.438			
PC O-38:4	0.365		0.395				
PC O-38:5		0.395					
PC O-38:6	0.478	0.348					
PC O-40:1							0.327
PC O-40:2	0.476	0.472				-0.315	
PC O-40:3	0.387	0.342					
PC O-40:4							
PC O-40:5		0.382					
PC O-40:6	0.402	0.432					
PC O-42:0							
PC O-42:2	0.312	0.455		0.418			
PC O-42:3		0.424		0.408			
PC O-42:4		0.341					
PC O-42:5							
PC O-44:3		0.432					
PC O-44:4					0.338		
PC O-44:5							
PC O-44:6							

-0.7 … 0.7

Supplementary Figure 2. Heatmap of partial Spearman correlation coefficients between changes in the blood clinical chemistry and metabolite levels obtained with a personalised dietary advice intervention. Analyses were controlled for age, sex and weight loss during the intervention. Coefficients are presented for significant correlations (p ≤0.05).
